# Word frequency modulates the use of initial and final morpheme positional probabilities during Chinese sentence reading in L2 learners: an eye-tracking study

**DOI:** 10.3389/fpsyg.2026.1916781

**Published:** 2026-08-17

**Authors:** Tingli Song, Ting Zhang

**Affiliations:** 1 School of Chinese Language and Literature, Nanjing Normal University, Nanjing, China; 2 Department of Chinese Language and Literature, Sun Yat-sen University, Guangzhou, China

**Keywords:** morpheme positional probability, word frequency, lexical recognition, word segmentation, L2 learners of Chinese, eye movements

## Abstract

**Introduction:**

Chinese text does not mark word boundaries with spaces, requiring readers to rely on statistical cues within character strings for word segmentation and lexical recognition. Morpheme positional probability (MPP), defined as the likelihood that a morpheme occurs in the initial or final position of a two-character word, is one such cue. However, it remains unclear how L2 learners of Chinese use initial and final MPP and whether this use is modulated by whole-word frequency.

**Methods:**

Using a controlled sentence-reading eye-tracking task, this study compared native Chinese readers, intermediate L2 learners, and advanced L2 learners during the processing of high- and low-frequency two-character words in which initial and final MPP were manipulated. Eye-movement data from 76 participants were analyzed using cross-material linear mixed-effects models.

**Results:**

The results showed significant reader-group effects across first fixation duration, gaze duration, go-past time, and total viewing time. Word frequency significantly affected gaze duration, go-past time, and total viewing time and significantly modulated the effect of initial MPP. The interaction between initial and final MPP varied across frequency conditions: in low-frequency words, initial MPP showed a more stable effect, whereas final MPP mainly influenced processing through its interaction with initial MPP.

**Discussion:**

These findings suggest that MPP functions as an important statistical cue in Chinese word segmentation and recognition, but its contribution is conditional on word frequency, processing stage, and cue configuration. L2 learners’ sustained sensitivity to MPP may reflect less automatized whole-word representations and greater reliance on sublexical processing. Although the present study examined online lexical processing rather than durable vocabulary learning, the findings provide indirect evidence for understanding the development of L2 Chinese lexical representations.

## 1 Introduction

Chinese text is written without explicit spaces between words. Readers must therefore segment continuous character strings, identify lexical units, and access meaning in a highly integrated manner. Chinese word segmentation and word recognition have been argued to operate as mutually constrained processes rather than as two independent stages (Li et al., 2009). From the perspective of visual word recognition, readers must encode both the identity of the morphemic constituents of a word and their relative positions within that word (Hua et al., 2017). Because two-character words are highly prevalent in modern Chinese (Beijing Language Institute Language Teaching Research Institute, 1986), the distribution of morphemes in word-initial and word-final positions is relatively regular. Lexical recognition in Chinese may therefore depend not only on morphemic meaning and frequency but also on the probability with which a morpheme occurs in a particular intraword position (Gu and Li, 2015).

Morpheme positional probability (MPP) refers to the statistical tendency for a morpheme to occur in a specific position within a two-character word. Initial MPP and final MPP can be calculated by dividing the frequency with which a morpheme appears as the first or second constituent of a two-character word by the total frequency with which that morpheme appears across possible two-character word positions (Cao et al., 2022). Because the characters in the present two-character compound words function as morphemic units, the term MPP is used throughout this article. MPP indexes the position-specific combinatorial strength of a character or morpheme and can be regarded as an implicit distributional cue acquired from long-term reading experience. More broadly, research on Chinese reading has shown that readers can exploit statistical regularities in character sequences to infer word boundaries during sentence processing (Yen et al., 2012). Previous work has shown that MPP supports word-boundary detection, word segmentation, and lexical identification in Chinese reading (Liang et al., 2022a,b, 2024).

The present study is framed within an interactive account of Chinese lexical processing. Following the integrated model of word processing and eye-movement control proposed by Li and Pollatsek (2020), Chinese word identification is assumed to involve parallel and mutually constraining activation of character, morpheme, and whole-word representations. Strong and automatized whole-word representations can provide relatively direct access to lexical-semantic information, whereas morphemic and character-level representations can support segmentation and lexical access when whole-word familiarity is weak or when word boundaries are uncertain. In this framework, representational strength refers to the efficiency, consistency, and automaticity with which a lexical form activates meaning and competes with alternative segmentations. MPP functions as a position-specific prior: it biases candidate-word activation, helps readers evaluate possible word boundaries, and influences the relative weighting of whole-word and morpheme-based processing routes. Native and L2 systems are therefore not assumed to differ categorically. Rather, they are expected to differ quantitatively in the automatization of whole-word representations and in the weight assigned to sublexical statistical cues during online reading.

However, the relative contributions and time courses of initial and final MPP remain unsettled. One source of inconsistency is that previous studies differ in task demands, age or language-experience groups, and whether the relevant cue is available in foveal or parafoveal processing. For example, Cui et al. (2013) used an eye-movement-contingent display-change paradigm to examine how constituent characters contribute to compound-word processing and found stronger reliance on the first constituent.Using overlapping ambiguous strings, Ma et al. (2014) further showed that word segmentation during Chinese reading is influenced by competing lexical candidates, highlighting the dynamic interaction between constituent information and whole-word processing. Gao (2018), using lexical decision and naming tasks, reported that both children and adults could use initial and final positional probability information, although adults showed more efficient use. Li et al. (2025), using a masked lexical-decision paradigm, concluded that both initial and final MPP affected two-character word recognition, but with different time courses in position coding. By contrast, Liang et al. (2022a,b), using natural sentence reading and parafoveal-processing manipulations, found robust effects of final MPP but less reliable effects of initial MPP. Liang et al. (2024) later found that both initial and final MPP influenced incidental word learning during reading and that initial MPP showed an earlier and more stable effect. These apparently inconsistent findings may reflect differences in stimulus familiarity, task sensitivity, and the extent to which whole-word representations reduce the need for sublexical cue use. Word frequency is therefore a plausible moderator of MPP effects. Recent evidence from novel word learning further suggests that children can use both initial and final character positional probabilities to support word segmentation and recognition during reading, with the initial-position effect emerging earlier (Feng et al., 2026). Taken together, these findings indicate that positional probability cues may support not only the online recognition of familiar words but also the early formation of lexical representations.

As noted above, word frequency, expressed in the present framework as the strength of whole-word familiarity, is a central determinant of lexical recognition (Li and Pollatsek, 2020). Low-frequency words generally require more processing effort than high-frequency words, as reflected in longer fixation durations and greater processing load (Zang et al., 2012). Consistent with the interactive account introduced earlier, the frequency effect is commonly interpreted in terms of representational strength: high-frequency words are encountered more often and are more likely to develop stable whole-word representations, whereas low-frequency words have weaker representations and may require greater reliance on morphemic decomposition and other sublexical cues (Bai et al., 2015). A central question in Chinese psycholinguistics is therefore whether compound words are accessed primarily through whole-word representations, through morphemic representations, or through an interaction between the two. Cao et al. (2023) showed that whole-word frequency modulates MPP effects: high-frequency words, due to stronger whole-word representations, place reduced demands on morphemic decomposition, whereas low-frequency words rely more on MPP to support sublexical form-to-meaning mapping.

Recent studies of natural Chinese reading further indicate that whole-word frequency and sublexical constituent frequency jointly shape lexical access. Fu et al. (2025), controlling for stroke complexity while manipulating word length and word frequency, found that higher-frequency words produced shorter fixation durations across several eye-movement measures, whereas word length and word frequency contributed differently to lexical identification and saccade targeting. Zeng et al. (2025), using co-registration of eye movements and EEG, reported that whole-word frequency shortened fixation times and modulated character-frequency effects, indicating that constituent frequency effects are not independent of the familiarity of the whole word. A recent meta-analysis of Chinese compound word recognition also showed that constituent effects are robust across studies, supporting the view that whole-word access and morphemic decomposition jointly contribute to lexical identification (Zhang et al., 2026). Examining word frequency together with initial and final MPP is therefore necessary for clarifying the dynamic relationship between whole-word familiarity and sublexical statistical cues.

For L2 learners of Chinese, the use of MPP may be especially sensitive to language experience and the quality of lexical representations. Gao (2018) found that native-speaking children used MPP less efficiently than native-speaking adults, suggesting that this implicit statistical cue develops with reading experience. This developmental claim does not necessarily predict that native adults should show the largest observable MPP effects in eye movements. Increased exposure may yield more reliable knowledge of positional probabilities, while at the same time strengthening whole-word representations and reducing the need for overt reliance on MPP during online reading. L2 learners may have sufficient exposure to extract coarse position-specific regularities, but their whole-word access may be less automatized. Their eye movements may therefore remain more sensitive to MPP, not because their statistical knowledge is necessarily more accurate than that of native readers, but because they assign greater online weight to sublexical cues.

Evidence from L2 Chinese readers remains limited. Li et al. (2024) examined Uyghur college students learning Chinese and compared words with high initial and high final MPP against words with low initial and low final MPP. The high-probability condition yielded shorter fixation times, indicating that Uyghur learners with substantial L2 Chinese reading experience were sensitive to MPP during sentence reading. Lu and Huang (2023), using a lexical naming task, showed that the MPP of a specific character in multisyllabic words affected word segmentation in advanced L2 learners. Xing and Jiang (2018) further reported that the speed with which L2 learners extracted morpheme position information increased from the elementary to the intermediate level. These findings suggest that L2 learners can use morpheme positional information, but they do not clarify how learners at different proficiency levels use initial and final MPP, or how word frequency modulates this process.

Related research also indicates that L2 Chinese word segmentation difficulty is associated with experience with unspaced writing, transfer of processing strategies, and the quality of lexical representations. Cui (2023) found that interword spacing reduced fixation counts and regressions in L2 Chinese reading, suggesting reduced processing load during word-boundary identification. Yao et al. (2025) directly examined whether L2 word-segmentation difficulty reflects a processing strategy or a representational difference. They showed that advanced L2 learners could process high-frequency two-character compounds as whole units when word boundaries were explicitly marked by interword spaces, but their processing was disrupted under intercharacter spacing. Here, first-language spacing strategies refer to the expectation, learned from writing systems with visual word-boundary markers, that spaces provide reliable segmentation cues. Zhang et al. (2021) tested L2 Chinese reading comprehension using mediation models and found that morphological awareness and written vocabulary knowledge contributed to comprehension through lexical inference. These findings suggest that, relative to native readers, even advanced L2 learners may possess some whole-word representations while still depending more heavily on explicit boundary cues, morphological awareness, or sublexical decomposition during online segmentation.

In summary, previous studies have established MPP as an important statistical cue for Chinese word segmentation and lexical recognition, and the L2 evidence reviewed above further indicates that learners’ segmentation difficulty is shaped by writing-system experience and the quality of their lexical representations. Three questions, however, remain unresolved. First, the relative roles and time courses of initial and final MPP are still debated, and the inconsistencies may be partly due to differences in word frequency and stimulus familiarity. Second, few studies have examined whole-word frequency and initial and final MPP simultaneously, making it difficult to determine how representational strength modulates the use of sublexical statistical cues. Third, most available evidence comes from native readers, and little is known about whether learners at different levels of L2 Chinese proficiency use MPP differently under high- and low-frequency conditions.

The present study used eye tracking in a controlled sentence-reading task to manipulate word frequency, initial MPP, and final MPP, and to compare lexical processing in native Chinese readers, intermediate L2 learners, and advanced L2 learners. Based on the studies reviewed above, we formulated three expectations. First, both initial and final MPP were expected to influence target-word processing because both cues have been shown to support Chinese word segmentation and identification. However, initial MPP was expected to show a more stable effect because word-initial constituents often receive earlier attentional and lexical activation in left-to-right reading (Cui et al., 2013; Cao et al., 2023; Liang et al., 2024; Feng et al., 2026). Second, word frequency was expected to modulate MPP effects, with high-frequency words relying more on whole-word access and low-frequency words relying more on sublexical statistical cues. Third, because L2 learners may have less automatized whole-word representations, they were expected to show stronger observable reliance on MPP than native readers, although this reliance was expected to decrease with proficiency.

## 2 Materials and methods

### 2.1 Participants

The final sample included 76 valid participants. The native Chinese readers were university students. The L2 learners came from diverse first-language backgrounds, including English, French, Turkish, Arabic, Bengali, Urdu, Turkmen, Russian, Spanish, and Kyrgyz. Because these writing systems differ in word-boundary marking, writing direction, and orthographic depth, the L2 sample is described here as having experience with writing systems that provide explicit word-boundary conventions rather than as a homogeneous alphabetic-background group. Participants were recruited through university language programs, course announcements, and campus advertisements. Inclusion criteria were normal or corrected-to-normal vision, no self-reported reading disorder or neurological disorder, and sufficient familiarity with simplified Chinese characters to complete the sentence-reading task. Participants whose eye movements could not be calibrated reliably were excluded from analysis.

The L2 learners were initially divided into intermediate and advanced groups according to their HSK scores. HSK refers to Hanyu Shuiping Kaoshi, the standardized Chinese Proficiency Test for non-native speakers. Learners with HSK 4, or HSK 5 scores below 210, were assigned to the intermediate group. Learners with HSK 5 scores above 210 or HSK 6 were assigned to the advanced group. All participants completed a language-background questionnaire and a 30-item cloze test of Chinese proficiency (Feng et al., 2020). The wider age range in the L2 groups reflected the inclusion of both university language-program students and postgraduate students. Age information is reported descriptively in Table 1.

**Table 1:** Participant characteristics for the final eye-movement sample.

Group	*N*	Gender (M/F)	Age M (SD), range	Months learning Chinese M (SD), range	Chinese proficiency M (SD), range
Native speakers	31	11/20	23.16 (2.05), 18–28	N/A	28.06 (1.63), 23–30
Advanced L2 learners	23	6/17	25.43 (4.26), 18–38	67.57 (37.26), 12–168	26.26 (1.94), 22–29
Intermediate L2 learners	22	11/11	25.50 (4.19), 19–35	24.18 (20.02), 10–72	14.14 (3.75), 8–19

The final eye-movement analyses included 31 native speakers, 23 advanced L2 learners, and 22 intermediate L2 learners. The advanced and intermediate L2 groups differed significantly in the length of Chinese learning, Welch’s *t*(34.03) = −4.89, *p* < 0.001. The three groups also differed significantly in Chinese proficiency scores, *F*(2, 73) = 219.70, *p* < 0.001. Tukey *post hoc* comparisons showed significant differences between all pairs of groups (ps < 0.05). Participant characteristics for the final eye-movement sample are shown in Table 1.

### 2.2 Design

The study adopted a 3 (reader group: native speakers, intermediate L2 learners, advanced L2 learners) × 2 (word frequency: high, low) × 2 (initial MPP: high, low) × 2 (final MPP: high, low) mixed design. Reader group was a between-participants factor, whereas word frequency, initial MPP, and final MPP were within-participants factors. High- and low-frequency words formed two stimulus sets. The cross-material combined model served as the primary analysis because it directly tested whether word frequency modulated the effects of initial and final MPP. Stimulus-set-specific analyses were then conducted as follow-up analyses to clarify the high- and low-frequency patterns.

### 2.3 Stimuli

All target stimuli were two-character words selected from the vocabulary list of the Chinese Proficiency Grading Standards for International Chinese Language Education (hereafter, the Grading Standards). We first segmented all 7,976 two-character words in the list and extracted 2,886 co-occurring morphemic constituents. To ensure that positional probabilities were never estimated from a single word, only morphemes that occurred in at least two different two-character compound words in the vocabulary list were retained for the MPP computation. We then counted the number of two-character words in which each morpheme participated, the frequency with which each morpheme occurred in word-initial and word-final positions, and finally computed initial and final MPP. Following previous work, morphemes with positional probabilities above 0.70 were defined as high-probability morphemes, whereas those below 0.30 were defined as low-probability morphemes.

Word frequency was operationalized using the vocabulary levels in the Grading Standards, supplemented by frequency information from SUBTLEX-CH (Cai and Brysbaert, 2010). Words from Levels 1–5 were assigned to the high-frequency stimulus set, and words from Levels 6–9 to the low-frequency stimulus set. The Grading Standards levels are not identical to continuous corpus-based frequency measures in the psycholinguistic sense, because they also reflect instructional sequencing, lexical difficulty, familiarity, and usage contexts. The present study therefore treats this classification as a categorical proxy for word frequency and learning familiarity, and this limitation is addressed in the Discussion.

The high-frequency set consisted of 40 target words, with 10 words in each initial-final MPP combination (HH, HL, LH, and LL). The low-frequency set consisted of 32 target words, with 8 words in each combination. Material-control analyses were conducted separately for the high- and low-frequency sets. Across the four MPP conditions, the target words did not differ significantly in initial character frequency, final character frequency, word frequency, initial character stroke count, or final character stroke count (high-frequency set: Fs < 2.76, ps > 0.05; low-frequency set: Fs < = 1.75, ps > = 0.180). Descriptive statistics for the stimuli are shown in Tables 2, 3. Although these tests indicated no reliable condition-level differences, item means, especially corpus word frequency in the high-frequency set, still varied across MPP combinations. The mixed-effects models therefore included item random intercepts to account for item-level variability. Because the number of items was limited, fully stable models including all item-level covariates could not be fitted. This limitation is addressed in the Discussion.

**Table 2:** Characteristics of high-frequency target words.

Condition	Initial character frequency M (SD)	Final character frequency M (SD)	Word frequency M (SD)	Initial strokes M (SD)	Final strokes M (SD)
HH	896.89 (791.03)	2550.60 (3127.70)	240.64 (286.46)	8.00 (3.33)	6.60 (2.22)
HL	1426.43 (1193.84)	1968.53 (1738.96)	393.65 (587.99)	5.60 (1.84)	5.90 (1.91)
LH	442.55 (514.41)	1138.39 (1477.28)	61.98 (79.84)	8.50 (2.95)	9.20 (3.01)
LL	1113.42 (1679.14)	1356.49 (1551.45)	16.92 (16.36)	8.00 (4.42)	7.30 (3.83)

**Table 3:** Characteristics of low-frequency target words.

Condition	Initial character frequency M (SD)	Final character frequency M (SD)	Word frequency M (SD)	Initial strokes M (SD)	Final strokes M (SD)
HH	157.00 (130.00)	510.00 (915.00)	1.31 (1.57)	8.25 (2.92)	7.38 (1.69)
HL	517.00 (657.00)	665.00 (997.00)	1.24 (0.86)	8.38 (2.83)	7.50 (3.46)
LH	666.00 (678.00)	316.00 (312.00)	2.88 (2.17)	7.12 (4.52)	7.75 (2.82)
LL	614.00 (875.00)	1260.00 (2235.00)	2.50 (2.18)	6.75 (2.60)	7.88 (3.44)

To further control stimulus properties, sentence difficulty, sentence naturalness, and contextual predictability were independently rated. Because all targets were two-character words embedded in short sentences, target position and sentence length were kept relatively stable during stimulus construction. Semantic transparency, age of acquisition, and lexical neighborhood measures were not separately quantified. This limitation is discussed below.

All targets were embedded in controlled sentences. Sentences in the high-frequency set were 11–15 Chinese characters long, and the same construction principles were used for the low-frequency set. To ensure that L2 learners could understand the sentence contexts, non-target words were kept, whenever possible, within Level 4 of the Grading Standards. Fifteen native speakers who did not participate in the eye-tracking experiment rated sentence difficulty and naturalness on 5-point scales. The mean difficulty rating was 1.82 (SD = 0.42) for the high-frequency set and 1.73 (SD = 0.32) for the low-frequency set. The difference was not significant, Welch’s *t*(69.79) = 1.06, *p* = 0.293. The mean naturalness rating was 4.01 (SD = 0.32) for the high-frequency set and 4.20 (SD = 0.30) for the low-frequency set. Although the low-frequency set was rated slightly more natural, Welch’s *t*(68.41) = −2.72, *p* = 0.008, both sets received mean naturalness ratings above 4. A separate group of 15 native speakers rated contextual predictability using a cloze procedure. Responses that matched the target word were scored as 1, and all other responses as 0. Mean predictability was 0.015 (SD = 0.075) for the high-frequency set and 0.000 (SD = 0.000) for the low-frequency set, indicating that the targets were generally not predictable from the sentence frames. To reduce strategic processing, 40 filler sentences were included in the high-frequency set and 32 in the low-frequency set. In each set, 12 filler trials were followed by comprehension judgment questions.

Some of the experimental sentences, that is, sentences that were part of the tested materials presented to participants, used metalinguistic frames, such as ‘The teacher explained the grammatical features of X’ or ‘The teacher taught us the new word X.’ In these sentences, the target word was mentioned as a lexical item rather than used as a normal argument or predicate in discourse. These frames helped keep syntactic position and semantic context comparable across target words that differed in frequency and MPP. However, they may have increased attention to the orthographic form of the target. The task is therefore characterized as a controlled sentence-reading task rather than a fully naturalistic reading task.

### 2.4 Apparatus and procedure

Eye movements were recorded from the right eye using an SR Research EyeLink 1,000 Plus system at a sampling rate of 1,000 Hz. Stimuli were displayed on a monitor with a resolution of 1,024 × 768 pixels and a refresh rate of 140 Hz. Participants viewed the screen from a distance of approximately 60 cm. Sentences were presented in 20-point SimSun font. Each Chinese character occupied 24 × 24 pixels, corresponding to approximately 1.09 degrees of visual angle. Before the experiment, participants were instructed to read each sentence silently for comprehension at a natural pace and to answer occasional yes/no comprehension questions. The instructions did not mention target words, MPP, or word frequency. A three-point calibration and drift correction were then performed. Calibration error was below 0.5 degrees for all participants. Participants completed four practice trials before the formal experiment. In each trial, a fixation cross was presented for 500 ms on the left side of the screen at center height, and the sentence appeared once the participant fixated the cross. Sentences were presented one at a time on a single line. Participants pressed the spacebar to proceed. When a comprehension judgment question appeared, participants pressed F for ‘yes’ and J for ‘no.’ Target and filler trials were pseudo-randomized within each block, and the two frequency blocks were separated by a short break. A one-point calibration check was conducted every other sentence, and recalibration was performed when necessary. The experiment lasted approximately 35 min.

### 2.5 Eye-movement measures and preprocessing

The target word was defined as the interest area. Four eye-movement measures were analyzed: first fixation duration (FFD), gaze duration (GD), go-past time (GPT), and total viewing time (TVT). FFD was the duration of the first fixation on the target interest area and was taken to reflect early sensitivity to orthographic and lexical form. GD was the sum of all fixations from the first fixation on the target until the eyes first left the target and was interpreted as an index of early lexical identification and integration. GPT was the summed fixation time from first fixation on the target until the eyes first moved to the right of the target, reflecting lexical access and local semantic integration. TVT was the sum of all fixations on the target interest area and was taken to reflect later reanalysis and integration (Rayner, 1998; Yan et al., 2013).

Data preprocessing followed Rayner (2009). Target-word observations shorter than 80 ms were removed, as were trials with tracking loss or without fixation on the target. Observations more than +/− 2.5 SD from the group mean for a given measure were also removed. Target-word skipping trials were excluded from the analyses of FFD, GD, and GPT. For TVT, observations below 80 ms were likewise excluded. Because short-fixation removal, tracking loss, and target skipping were recorded as a combined basic-exclusion flag in the preprocessing output, the Results section reports this basic-exclusion rate separately from additional outlier trimming.

Comprehension questions were attached only to filler trials and did not correspond directly to target-word trials. Therefore, the main eye-movement analyses did not further exclude target trials according to comprehension accuracy. Robustness analyses based on comprehension accuracy are reported after the main models as supplementary checks.

### 2.6 Statistical analysis

All fixation-duration measures were natural-log transformed to reduce distributional skew. Fixed effects included reader group, word frequency, initial MPP, final MPP, and their interactions. Native speakers served as the reference level for reader group, high-frequency words as the reference level for frequency, and high initial and high final MPP as the reference levels for the MPP factors. Random effects included random intercepts for participants and items. The cross-material combined model was fitted to the trial-level data as logRT ~ Group × Frequency × InitialMPP × FinalMPP + (1|Participant) + (1|Item). Fixed effects were evaluated using Wald chi-square tests. Approximate marginal and conditional R2 values are reported as indices of model fit. *Post hoc* and robustness analyses used the same preprocessing criteria. Full fixed-effect estimates, standard errors, and 95% confidence intervals are provided in the Supplementary material.

### 2.7 Ethics statement

The study involving human participants was reviewed and approved by the Biomedical Research Ethics Committee of Nanjing Normal University (Approval No. NNU20251001). All participants were informed of the study purpose, experimental procedures, potential risks, and right to withdraw before the experiment and provided written informed consent.

## 3 Results

### 3.1 Comprehension accuracy and data exclusion

In the high-frequency set, comprehension accuracy was 98.66% (SD = 3.79%, range = 83.33–100%) for native speakers, 98.55% (SD = 3.23%, range = 91.67–100%) for advanced L2 learners, and 88.26% (SD = 12.77%, range = 50–100%) for intermediate L2 learners. In the low-frequency set, comprehension accuracy was 97.42% (SD = 4.45%, range = 90–100%) for native speakers, 93.04% (SD = 9.74%, range = 70–100%) for advanced L2 learners, and 69.09% (SD = 13.77%, range = 50–90%) for intermediate L2 learners.

Combined basic exclusions for the high-frequency set, including target observations shorter than 80 ms, tracking loss, no target fixation, or target skipping, accounted for 8.19% of observations for FFD and GD and 8.13% for GPT and TVT. Additional +/−2.5 SD outlier trimming removed 2.47% of FFD observations, 2.65% of GD observations, 2.69% of GPT observations, and 3.01% of TVT observations. The resulting total exclusion/missing-data rates were 10.46, 10.63, 10.59, and 10.89%, respectively. For the low-frequency set, combined basic exclusions accounted for 4.56% of observations across measures. Additional outlier trimming removed 2.76% of FFD observations, 2.67% of GD observations, 2.46% of GPT observations, and 3.10% of TVT observations. The resulting total exclusion/missing-data rates were 7.20, 7.11, 6.91, and 7.52%, respectively.

### 3.2 Cross-material combined model

Because the central research question concerned whether word frequency modulated MPP effects, the cross-material combined model is reported before the separate high- and low-frequency follow-up analyses. This model tested the effects of reader group, word frequency, initial MPP, final MPP, and their interactions across all target items. All four models converged. Model fit was as follows: FFD, marginal R2 = 0.038 and conditional R2 = 0.133; GD, marginal R2 = 0.256 and conditional R2 = 0.384; GPT, marginal R2 = 0.203 and conditional R2 = 0.480; TVT, marginal R2 = 0.252 and conditional R2 = 0.394. The theoretically central fixed effects are reported in Table 4.

**Table 4:** Cross-material mixed-effects model tests for theoretically central fixed effects.

Effect	FFD	GD	GPT	TVT
Group	9.86 (2), *p* = 0.007	108.88 (2), *p* < 0.001	77.83 (2), *p* < 0.001	80.88 (2), *p* < 0.001
Frequency	1.21 (1), *p* = 0.271	16.33 (1), *p* < 0.001	4.62 (1), *p* = 0.032	6.05 (1), *p* = 0.014
Initial MPP	0.03 (1), *p* = 0.858	0.38 (1), *p* = 0.536	4.71 (1), *p* = 0.030	0.57 (1), *p* = 0.449
Final MPP	0.00 (1), *p* = 0.946	0.00 (1), *p* = 0.946	1.31 (1), *p* = 0.252	0.21 (1), *p* = 0.645
Group × frequency	2.97 (2), *p* = 0.226	9.27 (2), *p* = 0.010	27.06 (2), *p* < 0.001	22.14 (2), *p* < 0.001
Frequency × initial MPP	0.00 (1), *p* = 0.987	3.94 (1), *p* = 0.047	15.49 (1), *p* < 0.001	5.83 (1), *p* = 0.016
Frequency × final MPP	0.02 (1), *p* = 0.877	1.72 (1), *p* = 0.190	2.39 (1), *p* = 0.122	1.87 (1), *p* = 0.171
Initial MPP × final MPP	0.00 (1), *p* = 0.984	0.00 (1), *p* = 0.974	0.97 (1), *p* = 0.325	0.07 (1), *p* = 0.797
Frequency × initial MPP × final MPP	0.09 (1), *p* = 0.762	0.76 (1), *p* = 0.384	4.65 (1), *p* = 0.031	1.06 (1), *p* = 0.304
Group × frequency × initial MPP × final MPP	1.33 (2), *p* = 0.513	1.35 (2), *p* = 0.510	4.79 (2), *p* = 0.091	2.33 (2), *p* = 0.312

Reader group was significant across all four measures. Word frequency significantly affected GD, GPT, and TVT. Crucially, the frequency × initial MPP interaction was significant for GD, GPT, and TVT, demonstrating that word frequency directly modulated the effect of initial MPP. The frequency × initial MPP × final MPP interaction was significant only for GPT, suggesting that frequency modulation of the coordinated use of initial and final MPP emerged mainly during later lexical access and local semantic integration. The four-way group × frequency × initial MPP × final MPP interaction did not reach significance in any measure. Thus, although the groups showed descriptive differences in frequency-dependent MPP patterns, these differences should not be interpreted as evidence for a robust four-factor interaction.

### 3.3 Follow-up analyses for high-frequency words

**Figure 1:**
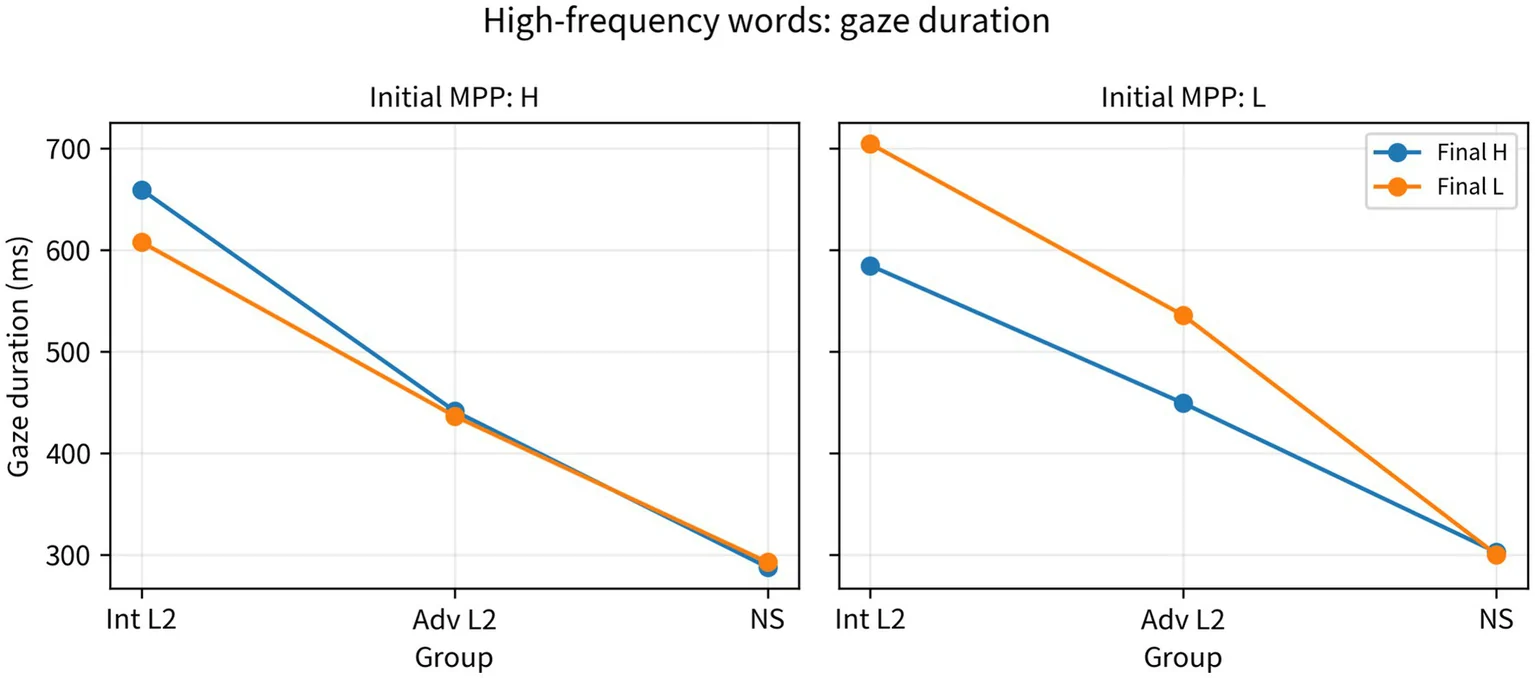
Gaze duration for high-frequency words as a function of group, initial morpheme positional probability, and final morpheme positional probability.

**Figure 2:**
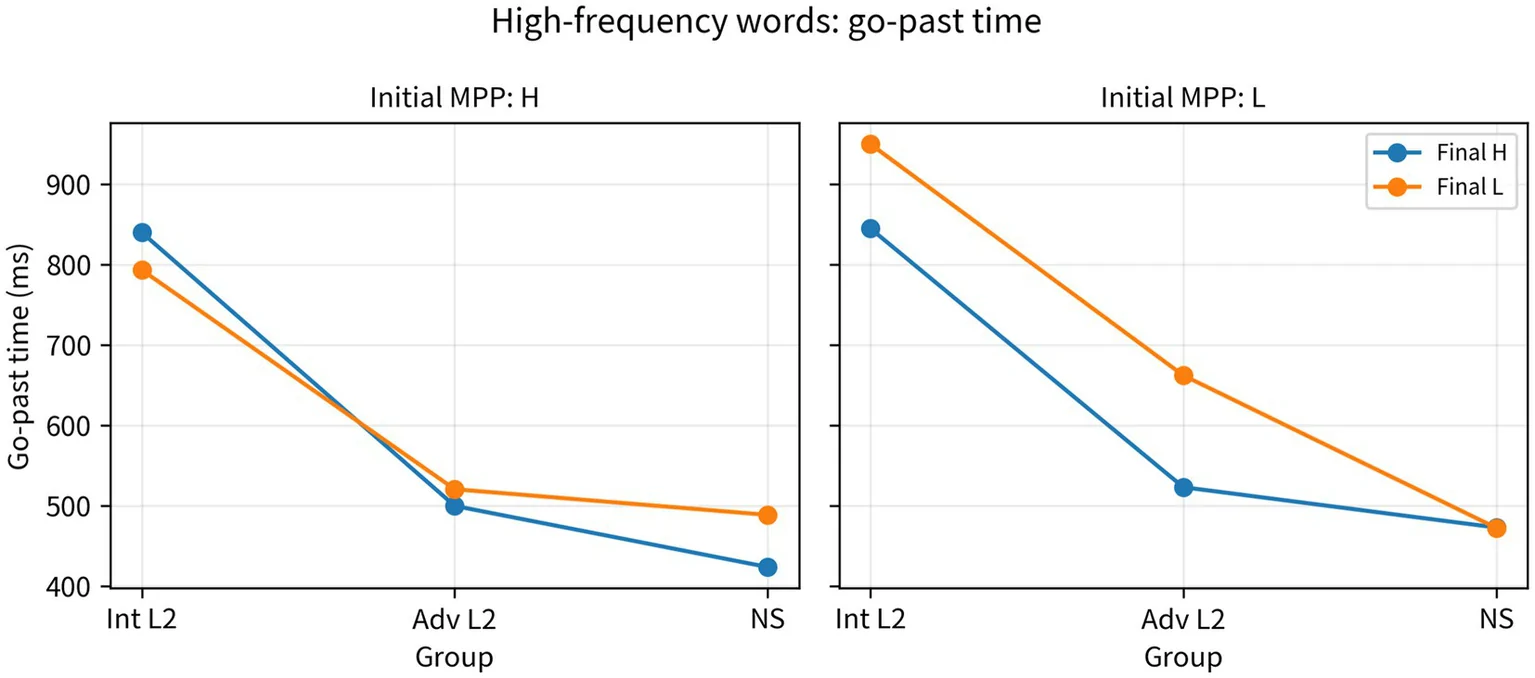
Go-past time for high-frequency words as a function of group, initial morpheme positional probability, and final morpheme positional probability.

**Figure 3:**
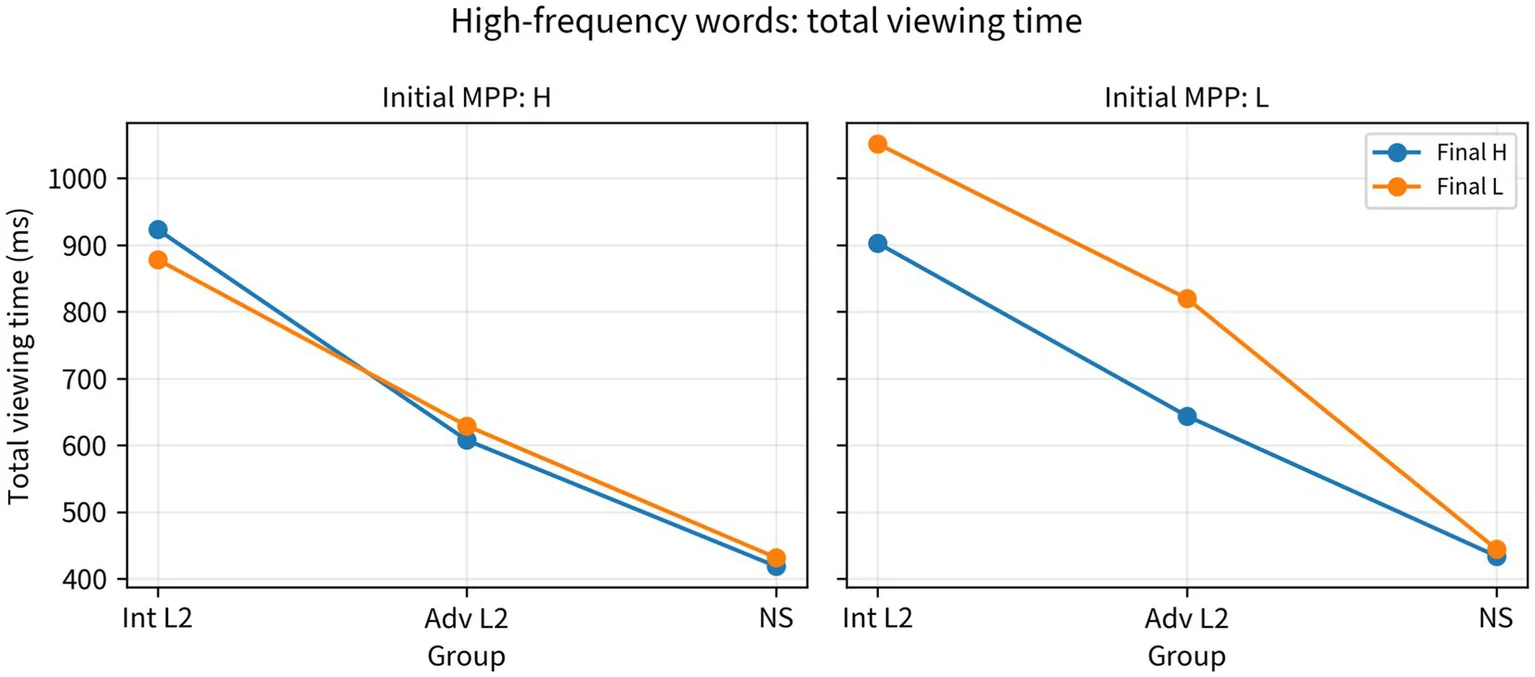
Total viewing time for high-frequency words as a function of group, initial morpheme positional probability, and final morpheme positional probability.

Descriptive statistics for the high-frequency set are shown in Table 5, and the interaction patterns are displayed in Figures 1–3. These analyses are reported as follow-up analyses to clarify the frequency-specific pattern observed in the combined model.

**Table 5:** Descriptive eye-movement measures for high-frequency words, M (SD).

Group	Initial MPP	Final MPP	FFD	GD	GPT	TVT
NS	H	H	243.27 (79.79)	287.54 (133.84)	423.52 (289.19)	418.69 (234.78)
NS	H	L	247.65 (89.21)	292.50 (135.84)	488.42 (358.88)	431.55 (270.72)
NS	L	H	245.87 (93.92)	302.34 (153.06)	472.74 (307.78)	433.96 (238.14)
NS	L	L	243.46 (82.92)	300.04 (145.29)	472.29 (319.29)	444.61 (237.84)
Adv L2	H	H	262.08 (82.05)	441.36 (208.16)	499.61 (266.79)	607.53 (363.43)
Adv L2	H	L	256.01 (73.76)	436.36 (226.08)	520.32 (316.40)	629.15 (372.16)
Adv L2	L	H	261.17 (77.22)	448.97 (202.93)	522.84 (269.19)	643.63 (349.49)
Adv L2	L	L	263.62 (82.18)	535.42 (280.69)	661.92 (358.85)	819.68 (413.62)
Int L2	H	H	282.00 (113.17)	659.15 (378.12)	839.82 (520.04)	923.19 (553.43)
Int L2	H	L	284.42 (97.57)	607.51 (363.48)	793.21 (528.48)	878.14 (586.77)
Int L2	L	H	286.87 (117.22)	584.42 (337.02)	844.89 (587.81)	902.64 (524.66)
Int L2	L	L	280.75 (109.77)	704.22 (432.24)	949.86 (604.56)	1051.43 (641.09)

NS, native speakers; Adv L2, advanced L2 learners; Int L2, intermediate L2 learners; H, high; L, low; FFD, first fixation duration; GD, gaze duration; GPT, go-past time; TVT, total viewing time.

For FFD, only the main effect of reader group was significant, chi-square(2) = 16.20, *p* < 0.001. Native speakers showed shorter FFDs than both L2 groups, and advanced L2 learners showed shorter FFDs than intermediate L2 learners. No other effect reached significance, suggesting that in the earliest fixation measure, group differences in processing efficiency were more prominent than MPP effects.

For GD, the main effect of reader group was significant, chi-square(2) = 77.99, *p* < 0.001. The main effect of initial MPP was also significant, chi-square(1) = 7.21, *p* = 0.007, and the main effect of final MPP was marginal, chi-square(1) = 3.72, *p* = 0.054. The interaction between initial and final MPP was significant, chi-square(1) = 11.57, *p* < 0.001, as was the three-way interaction among reader group, initial MPP, and final MPP, chi-square(2) = 7.78, *p* = 0.020 (Figure 1). Simple effects indicated that native readers were largely insensitive to MPP, whereas L2 learners showed facilitation from high initial MPP when final MPP was low. When initial MPP was low, both intermediate and advanced L2 learners showed facilitation from high final MPP.

For GPT, the main effects of reader group, chi-square(2) = 60.85, *p* < 0.001, initial MPP, chi-square(1) = 18.60, *p* < 0.001, and final MPP, chi-square(1) = 8.13, *p* = 0.004, were all significant. The three-way interaction was also significant, chi-square(2) = 12.36, *p* = 0.002 (Figure 2). TVT showed significant main effects of reader group, initial MPP, and final MPP, along with significant interactions involving reader group and the two MPP factors (Figure 3). Overall, in the high-frequency set, MPP effects were most evident among L2 learners and were more pronounced in GD, GPT, and TVT than in FFD.

### 3.4 Follow-up analyses for low-frequency words

**Figure 4:**
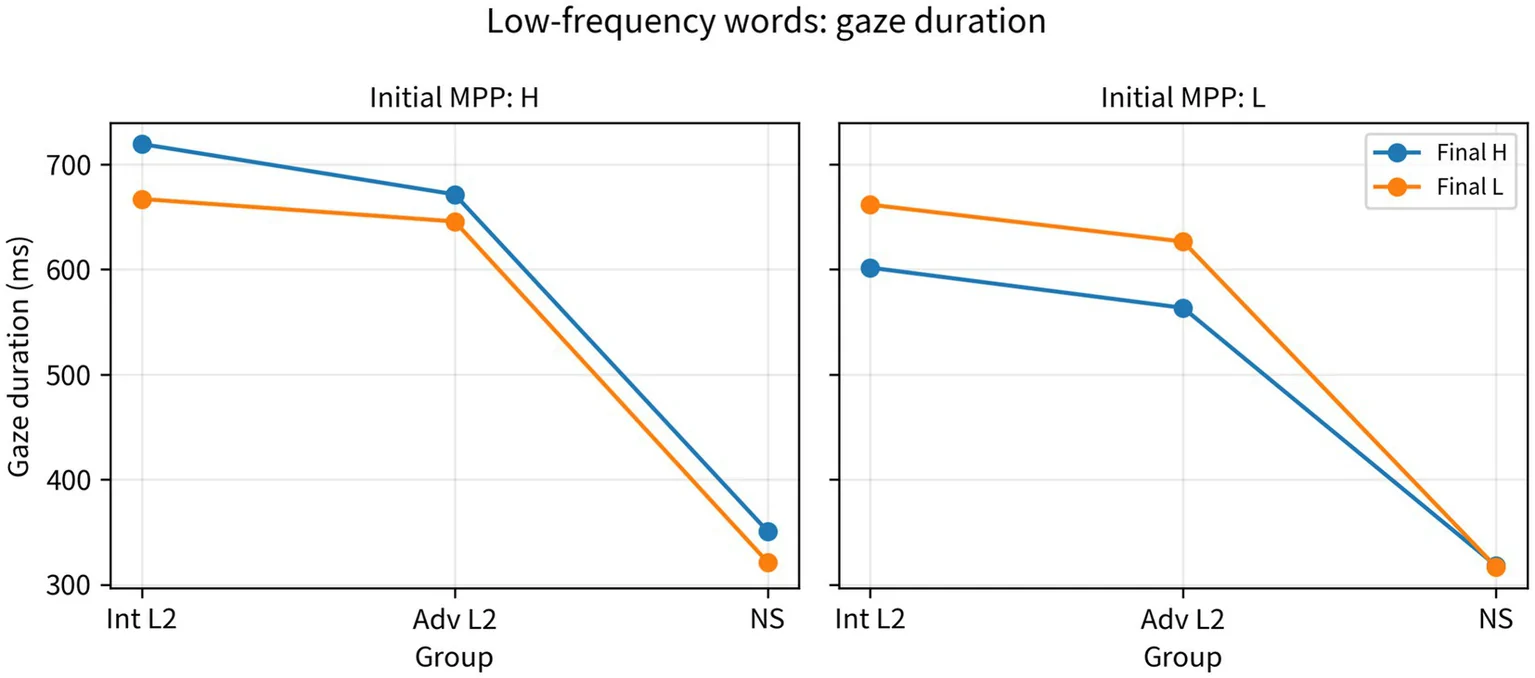
Gaze duration for low-frequency words as a function of group, initial morpheme positional probability, and final morpheme positional probability.

Descriptive statistics for the low-frequency set are shown in Table 6, and the GD pattern is displayed in Figure 4.

**Table 6:** Descriptive eye-movement measures for low-frequency words, M (SD).

Group	Initial MPP	Final MPP	FFD	GD	GPT	TVT
NS	H	H	249.51 (77.74)	350.71 (167.52)	441.16 (236.77)	457.61 (231.53)
NS	H	L	250.26 (84.27)	321.31 (151.84)	414.77 (235.15)	437.70 (243.59)
NS	L	H	248.98 (78.20)	317.89 (145.91)	369.40 (202.22)	408.76 (210.38)
NS	L	L	252.87 (79.67)	316.62 (150.11)	398.04 (222.46)	427.76 (224.18)
Adv L2	H	H	272.36 (86.67)	671.38 (344.58)	805.84 (411.41)	864.23 (431.52)
Adv L2	H	L	265.86 (90.86)	645.61 (314.43)	732.93 (377.78)	860.65 (424.64)
Adv L2	L	H	272.59 (90.06)	563.34 (304.09)	685.13 (422.99)	756.97 (411.22)
Adv L2	L	L	289.02 (99.23)	626.44 (329.00)	795.65 (473.89)	883.20 (475.66)
Int L2	H	H	316.48 (130.95)	719.33 (350.91)	881.90 (506.42)	892.10 (460.37)
Int L2	H	L	298.72 (111.79)	667.00 (373.35)	853.49 (529.50)	886.35 (497.34)
Int L2	L	H	288.60 (116.31)	601.63 (342.69)	845.97 (559.99)	816.27 (466.52)
Int L2	L	L	302.71 (113.10)	661.60 (328.67)	852.81 (445.26)	880.15 (469.81)

NS, native speakers; Adv L2, advanced L2 learners; Int L2, intermediate L2 learners; H, high; L, low; FFD, first fixation duration; GD, gaze duration; GPT, go-past time; TVT, total viewing time.

In the low-frequency set, FFD showed a significant main effect of reader group, chi-square(2) = 20.42, *p* < 0.001, and a significant interaction between initial and final MPP, chi-square(1) = 4.60, *p* = 0.032. The interaction was mainly reflected in a tendency for words with high final MPP to elicit longer FFDs than words with low final MPP within the low-initial-MPP condition.

For GD, the main effect of reader group was significant, chi-square(2) = 80.52, *p* < 0.001. The main effect of initial MPP was also significant, chi-square(1) = 18.12, *p* < 0.001, with higher initial MPP associated with longer GD. The initial MPP x final MPP interaction was significant, chi-square(1) = 11.00, *p* < 0.001 (Figure 4). Under the high-initial-MPP condition, words with low final MPP were processed faster than words with high final MPP. Under the low-initial-MPP condition, words with high final MPP were processed faster than words with low final MPP.

GPT and TVT showed similar patterns. For GPT, reader group, chi-square(2) = 71.94, *p* < 0.001, and initial MPP, chi-square(1) = 13.42, *p* < 0.001, showed significant main effects, and the initial MPP × final MPP interaction was significant, chi-square(1) = 14.34, *p* < 0.001. For TVT, the main effects of reader group, chi-square(2) = 65.81, *p* < 0.001, and initial MPP, chi-square(1) = 11.75, *p* < 0.001, were significant, as was the initial MPP × final MPP interaction, chi-square(1) = 8.79, *p* = 0.003. Unlike the high-frequency set, the low-frequency set did not show stable interactions involving reader group, suggesting that native and L2 readers may both rely more on sublexical statistical cues when processing low-frequency words, although L2 readers were generally slower.

### 3.5 Robustness analyses based on comprehension accuracy

Because the intermediate L2 group showed relatively low comprehension accuracy in the low-frequency set, we conducted robustness analyses in which participants with accuracy below 75 and 80% within a stimulus set were excluded. After these exclusions, the Frequency × Initial MPP effect remained significant in GD [75% threshold: chi-square(1) = 4.02, *p* = 0.045; 80% threshold: chi-square(1) = 4.09, *p* = 0.043], in GPT (chi-squares > 15.81, ps < 0.001), and in TVT (chi-squares > 5.88, ps < = 0.015). Thus, the central frequency-modulation effect was not driven by participants with low comprehension accuracy. These analyses were confirmatory and did not change the interpretation of the main model.

## 4 Discussion

### 4.1 MPP effects are conditional on frequency and processing stage

The present results do not support a simple, uniform MPP effect across all readers and all words. In the cross-material model, the only significant main effect of MPP was the effect of initial MPP in GPT. Final MPP did not show a reliable overall main effect. Instead, initial and final MPP contributed through interactions, especially interactions involving word frequency and, in the high-frequency follow-up analyses, reader group. Thus, the most appropriate conclusion is that MPP is a useful statistical cue for Chinese word segmentation and recognition, but its observable effect depends on word frequency, processing stage, and the relative informativeness of the initial and final constituents.

This conditional interpretation helps integrate previous mixed findings. Initial MPP showed a more stable contribution than final MPP, especially in GD, GPT, and TVT, and this pattern is consistent with Cao et al. (2023), Liang et al. (2024), and Feng et al. (2026). The word-initial position may receive greater attentional weight in left-to-right reading and may participate earlier in candidate-word activation and boundary prediction. Nevertheless, final MPP was not irrelevant. In several conditions it functioned as a confirmatory or compensatory cue, particularly when the initial cue was weak or when initial and final cues jointly constrained segmentation. Native readers showed only weak MPP sensitivity in the high-frequency set, suggesting that stable whole-word representations can reduce the need for overt reliance on positional-probability cues. In contrast, L2 readers showed strong MPP sensitivity in the same set, indicating greater reliance on sublexical cues during online processing.

### 4.2 Frequency-dependent reliance on sublexical statistical cues

The high- and low-frequency stimulus sets showed different MPP patterns. In the high-frequency set, higher MPP generally facilitated processing for L2 readers, especially when one constituent provided a strong cue and the other did not. In the low-frequency set, however, the direction of the initial-MPP effect changed: high initial MPP was associated with longer GD, GPT, and TVT. Thus, the term ‘MPP effect’ should not be interpreted as a single facilitative effect. High positional probability can facilitate processing when it confirms a familiar whole-word candidate, but it can increase processing cost when strong positional expectations conflict with weak whole-word familiarity or when additional morphemic integration is required.

One possible account is that high-frequency words have stronger whole-word representations, allowing readers to activate lexical information relatively quickly. Under such conditions, MPP may function mainly as a boundary-confirmation or processing-efficiency cue. Low-frequency words have weaker whole-word representations and require more bottom-up sublexical information. When a highly predictive initial morpheme occurs in a low-frequency word with relatively weak whole-word familiarity, the mismatch between strong positional expectation and weak lexical familiarity may increase integration cost. This interpretation is consistent with Cao et al.'s (2023) finding that word frequency modulates MPP effects and with Yu et al.'s (2021) account that word and character familiarity jointly affect segmentation in Chinese reading.

The cross-material combined model further supports the frequency-modulation account. The Frequency x Initial MPP interaction was significant in GD, GPT, and TVT, indicating that the effect of initial MPP changed as a function of whole-word frequency or familiarity. The Frequency x Initial MPP x Final MPP interaction was significant only in GPT, suggesting that the coordinated use of initial and final MPP was modulated by frequency mainly during later lexical access and local semantic integration. Because the four-way interaction was not significant, differences between native and L2 readers in the frequency-modulated MPP pattern should be interpreted cautiously.

A seemingly counterintuitive descriptive pattern was that, for intermediate learners, LL high-frequency words sometimes produced longer GD, GPT, and TVT than LL low-frequency words. We do not interpret this descriptive reversal as evidence that rarer words are easier for less experienced readers. The LL high-frequency items had relatively low corpus-frequency means within the high-frequency set and were embedded in a different stimulus set from the low-frequency items. In addition, sentence naturalness was slightly higher for the low-frequency set. The cross-material model, rather than individual cell means, is therefore the appropriate basis for inference about frequency modulation.

### 4.3 L2 readers: processing efficiency, not categorical representational difference

All three reader groups showed stable differences in processing efficiency across the main eye-movement measures: native speakers were generally faster than advanced L2 learners, who were in turn faster than intermediate L2 learners. This pattern indicates that lexical processing in L2 Chinese was less automatized than in native Chinese. In the high-frequency set, native readers were generally insensitive, or only weakly sensitive, to MPP, whereas L2 learners showed stronger MPP effects. This suggests that even when processing relatively familiar words, L2 readers may rely more heavily on intraword statistical cues and morphemic decomposition.

The comparison between intermediate and advanced L2 learners should be interpreted cautiously. Advanced learners were generally faster than intermediate learners, indicating greater processing efficiency. However, the cross-material four-way interaction involving group, frequency, initial MPP, and final MPP was not significant, and the follow-up analyses did not provide robust evidence that the two L2 groups used qualitatively different MPP strategies. The proficiency effect in the present data therefore appears to be primarily an efficiency difference rather than a clear difference in the structure of the MPP-based processing strategy.

This efficiency-based interpretation of the native-L2 contrast accords with recent studies of L2 Chinese word segmentation. Cui (2023) found that interword spacing reduced fixation and regression load in L2 Chinese reading. Yao et al. (2025) further showed that advanced L2 learners could process high-frequency compounds holistically when word boundaries were explicitly marked but remained influenced by L1 spacing strategies under intercharacter spacing conditions. These findings suggest that L2 readers do not lack whole-word representations altogether. Rather, their online use of such representations may be less automatic and less stable than that of native readers, making them more likely to fall back on character- or morpheme-based decomposition. Accordingly, the present study does not characterize the native-L2 contrast as a categorical or essential difference. Instead, it is more appropriately interpreted as a quantitative difference in representational automatization and in the relative weighting of whole-word and sublexical processing strategies.

### 4.4 Eye-movement time course of MPP effects

The four eye-movement measures further clarify the time course of MPP use. FFD showed primarily group differences and little evidence of MPP modulation, suggesting that MPP did not strongly affect the earliest fixation on the target word. In contrast, GD, GPT, and TVT showed more consistent frequency-dependent initial-MPP effects. This pattern suggests that MPP contributes mainly after initial visual uptake, during lexical identification, integration, and possible reanalysis. The emergence of the Frequency × Initial MPP × Final MPP interaction only in GPT further suggests that the coordinated use of initial and final positional cues was most evident when readers integrated the target word with the local sentence context and resolved segmentation or lexical-access difficulty.

### 4.5 Limitations and future directions

Several limitations should be noted. First, the present experiment measured online lexical processing during sentence reading rather than long-term vocabulary learning. That is, online lexical processing is contrasted here with durable word learning or retention, not with sentence reading itself. Although MPP may provide cues for novel word segmentation and lexical-representation formation, the study did not include novel word learning, delayed retention, or word-meaning tests. Therefore, it cannot directly demonstrate vocabulary acquisition effects. Second, word frequency was partly operationalized through the vocabulary levels of the Grading Standards. Although these levels are related to corpus frequency, they also reflect instructional sequencing, familiarity, and lexical difficulty. Future research should use continuous corpus-based frequency measures and additionally control or measure semantic transparency, familiarity, and age of acquisition. Contextual predictability was controlled in the present study through cloze ratings, but future work should continue to control it in larger stimulus sets. Third, although item random intercepts were included, the limited number of items made it difficult to include all item-level covariates in stable models. Replication with larger and more tightly matched item sets would allow continuous covariate models to be tested more directly. Fourth, the L2 sample was heterogeneous in first-language background, and the sample size did not allow systematic comparison across writing-system backgrounds. Future work should compare learners with alphabetic, abjad, and non-segmented writing-system experience. Such a comparison is theoretically motivated, because writing systems differ in whether word boundaries are visually marked, and first-language orthographic experience may shape the segmentation routines that readers transfer to L2 Chinese reading, thereby modulating the weight assigned to sublexical statistical cues such as MPP (Cui, 2023; Yao et al., 2025). Fifth, some target sentences used metalinguistic frames, which may have increased attention to target-word form. Future studies should use more natural sentence contexts or compare metalinguistic and natural frames directly. Finally, the number of low-frequency items in each condition was limited. Replication with larger item sets, open materials, open data, and preregistered analyses would strengthen the generalizability of the findings.

## 5 Conclusion

Using a controlled sentence-reading eye-tracking task, this study examined how word frequency, initial MPP, and final MPP affected lexical processing in native Chinese readers and intermediate and advanced L2 learners of Chinese. The results show that MPP is an important statistical cue in Chinese word segmentation and recognition, but its effects are conditional rather than uniformly facilitative. Cross-material models showed that word frequency significantly modulated the effect of initial MPP in GD, GPT, and TVT, indicating that whole-word frequency or familiarity changes readers’ reliance on sublexical statistical cues. High-MPP cues tended to facilitate processing in high-frequency words but were associated with increased processing costs in some low-frequency conditions. Compared with native speakers, L2 learners processed words more slowly and relied more strongly on MPP in the high-frequency condition, possibly reflecting weaker automatization of whole-word representations and continued involvement of morphemic decomposition. Future studies should use continuous frequency measures, more natural sentence contexts, and direct vocabulary-learning tests to further examine how MPP contributes to the development of L2 Chinese lexical representations.

## Data Availability

The original contributions presented in the study are included in the article/Supplementary material, further inquiries can be directed to the corresponding author.
