# Supplementary material for: Word frequency modulates the use of initial and final morpheme positional probabilities during Chinese sentence reading in L2 learners: an eye-tracking study
Source: Front Psychol. 2026 Aug 17;17:1916781. doi: 10.3389/fpsyg.2026.1916781 (PMC13526546; doi:10.3389/fpsyg.2026.1916781)
Supplement: Supplementary file 1 [file Table_1.DOCX]

Supplementary Material

Word frequency modulates the use of initial and final morpheme positional probabilities during Chinese sentence reading in L2 learners

# Supplementary Table S1. High-frequency target words

| **HH** | **HL** | **LH** | **LL** |
| --- | --- | --- | --- |
| 书店 | 打开 | 饭店 | 长大 |
| 晚饭 | 安全 | 班长 | 播出 |
| 筷子 | 太阳 | 假期 | 餐饮 |
| 再见 | 分开 | 语言 | 划分 |
| 回来 | 因为 | 态度 | 立即 |
| 电话 | 东西 | 味道 | 台阶 |
| 休息 | 可爱 | 见面 | 恋爱 |
| 黑板 | 难过 | 假期 | 点燃 |
| 排队 | 公平 | 计划 | 为难 |

# Supplementary Table S2. Example high-frequency sentences

| **MPP type** | **Target word** | **Chinese sentence** | **English gloss** |
| --- | --- | --- | --- |
| HH | 回来 | 这节课老师讲解了回来的语法特点。 | In this class, the teacher explained the grammatical features of huilai. |
| HL | 因为 | 这节课老师讲解了因为的语法特点。 | In this class, the teacher explained the grammatical features of yinwei. |
| LH | 态度 | 这节课老师讲解了态度的语法特点。 | In this class, the teacher explained the grammatical features of taidu. |
| LL | 立即 | 这节课老师讲解了立即的语法特点。 | In this class, the teacher explained the grammatical features of liji. |

# Supplementary Table S3. Low-frequency target words

| **HH** | **HL** | **LH** | **LL** |
| --- | --- | --- | --- |
| 宣扬 | 刮风 | 话费 | 区分 |
| 防范 | 孝敬 | 格式 | 养老 |
| 站点 | 喜庆 | 缘分 | 久违 |
| 旋涡 | 壮大 | 长辈 | 点评 |
| 朝代 | 联网 | 片段 | 味精 |
| 内向 | 太极 | 静止 | 任命 |
| 归纳 | 高超 | 习俗 | 立功 |
| 香油 | 宠爱 | 力度 | 造就 |

# Supplementary Table S4. Example low-frequency sentences

| **MPP type** | **Target word** | **Chinese sentence** | **English gloss** |
| --- | --- | --- | --- |
| HH | 站点 | 汉语老师教给我们站点这个新词语。 | The Chinese teacher taught us the new word zhandian. |
| HL | 喜庆 | 汉语老师教给我们喜庆这个新词语。 | The Chinese teacher taught us the new word xiqing. |
| LH | 缘分 | 汉语老师教给我们缘分这个新词语。 | The Chinese teacher taught us the new word yuanfen. |
| LL | 久违 | 汉语老师教给我们久违这个新词语。 | The Chinese teacher taught us the new word jiuwei. |

# Supplementary Table S5. Descriptive statistics for material matching

| **Frequency** | **Condition** | **InitialCharFreq** | **FinalCharFreq** | **WordFreq** | **InitialStrokes** | **FinalStrokes** |
| --- | --- | --- | --- | --- | --- | --- |
| High | HH | 896.89 (791.03) | 2550.60 (3127.70) | 240.64 (286.46) | 8.00 (3.33) | 6.60 (2.22) |
| High | HL | 1426.43 (1193.84) | 1968.53 (1738.96) | 393.65 (587.99) | 5.60 (1.84) | 5.90 (1.91) |
| High | LH | 442.55 (514.41) | 1138.39 (1477.28) | 61.98 (79.84) | 8.50 (2.95) | 9.20 (3.01) |
| High | LL | 1113.42 (1679.14) | 1356.49 (1551.45) | 16.92 (16.36) | 8.00 (4.42) | 7.30 (3.83) |
| Low | HH | 125.40 (123.21) | 504.13 (918.04) | 0.17 (0.07) | 7.25 (3.01) | 7.50 (1.77) |
| Low | HL | 517.15 (656.56) | 665.47 (996.54) | 1.24 (0.86) | 8.38 (2.83) | 7.50 (3.46) |
| Low | LH | 665.51 (678.26) | 316.46 (312.35) | 2.88 (2.17) | 7.12 (4.52) | 7.75 (2.82) |
| Low | LL | 613.74 (874.75) | 1259.56 (2234.58) | 2.50 (2.18) | 6.75 (2.60) | 7.88 (3.44) |

# Supplementary Table S6. ANOVA tests for material matching

| **Frequency** | **Variable** | **F** | **p** |
| --- | --- | --- | --- |
| High | InitialCharFreq | 1.3306 | 0.2796 |
| High | FinalCharFreq | 0.9332 | 0.4347 |
| High | WordFreq | 2.7572 | 0.0564 |
| High | InitialStrokes | 1.5924 | 0.2082 |
| High | FinalStrokes | 2.4948 | 0.0754 |
| Low | InitialCharFreq | 1.1450 | 0.3481 |
| Low | FinalCharFreq | 0.7685 | 0.5214 |
| Low | WordFreq | 4.7952 | 0.0081 |
| Low | InitialStrokes | 0.3540 | 0.7865 |
| Low | FinalStrokes | 0.0322 | 0.9921 |

# Supplementary Table S7. Sentence difficulty and naturalness ratings by frequency set

| **Measure** | **Frequency** | **Grand M** | **Grand SD** | **Item M mean** | **Item M SD** | **N items** |
| --- | --- | --- | --- | --- | --- | --- |
| Difficulty | High | 1.8233 | 0.9969 | 1.8233 | 0.4204 | 40.0000 |
| Difficulty | Low | 1.7312 | 0.9233 | 1.7312 | 0.3170 | 32.0000 |
| Fluency | High | 4.0050 | 1.2292 | 4.0050 | 0.3211 | 40.0000 |
| Fluency | Low | 4.2042 | 1.2002 | 4.2042 | 0.2981 | 32.0000 |

# Supplementary Table S8. Contextual predictability by frequency set

| **Frequency** | **M** | **SD** | **Min** | **Max** | **N items** |
| --- | --- | --- | --- | --- | --- |
| High | 0.0150 | 0.0748 | 0.0000 | 0.4670 | 40.0000 |
| Low | 0.0000 | 0.0000 | 0.0000 | 0.0000 | 32.0000 |

# Supplementary Table S9. Comprehension accuracy by group and frequency set

| **Frequency** | **Group** | **M** | **SD** | **Min** | **Max** | **N** |
| --- | --- | --- | --- | --- | --- | --- |
| High | Native | 0.9866 | 0.0379 | 0.8333 | 1.0000 | 31.0000 |
| High | Advanced | 0.9855 | 0.0323 | 0.9167 | 1.0000 | 23.0000 |
| High | Intermediate | 0.8826 | 0.1277 | 0.5000 | 1.0000 | 22.0000 |
| High | L2 overall | 0.9352 | 0.1049 | 0.5000 | 1.0000 | 45.0000 |
| Low | Native | 0.9742 | 0.0445 | 0.9000 | 1.0000 | 31.0000 |
| Low | Advanced | 0.9304 | 0.0974 | 0.7000 | 1.0000 | 23.0000 |
| Low | Intermediate | 0.6909 | 0.1377 | 0.5000 | 1.0000 | 22.0000 |
| Low | L2 overall | 0.8133 | 0.1687 | 0.5000 | 1.0000 | 45.0000 |

# Supplementary Table S10. Data exclusion and missing rates

| **Frequency** | **Measure** | **total** | **after_basic** | **after_trim** | **removed_total_pct** | **removed_outlier_pct** |
| --- | --- | --- | --- | --- | --- | --- |
| High | FFD | 3040.00 | 2791.00 | 2722.00 | 10.4605 | 2.4722 |
| High | GD | 3040.00 | 2791.00 | 2717.00 | 10.6250 | 2.6514 |
| High | GPT | 3040.00 | 2793.00 | 2718.00 | 10.5921 | 2.6853 |
| High | TVT | 3040.00 | 2793.00 | 2709.00 | 10.8882 | 3.0075 |
| Low | FFD | 2432.00 | 2321.00 | 2257.00 | 7.1957 | 2.7574 |
| Low | GD | 2432.00 | 2321.00 | 2259.00 | 7.1135 | 2.6713 |
| Low | GPT | 2432.00 | 2321.00 | 2264.00 | 6.9079 | 2.4558 |
| Low | TVT | 2432.00 | 2321.00 | 2249.00 | 7.5247 | 3.1021 |

# Supplementary Table S11. Cross-material model term tests

| **Measure** | **Effect** | **Chi-square** | **df** | **p** |
| --- | --- | --- | --- | --- |
| FFD | Group | 9.8584 | 2.0000 | 0.0072 |
| FFD | Frequency | 1.2129 | 1.0000 | 0.2708 |
| FFD | Initial MPP | 0.0319 | 1.0000 | 0.8582 |
| FFD | Final MPP | 0.0046 | 1.0000 | 0.9460 |
| FFD | Group × Frequency | 2.9709 | 2.0000 | 0.2264 |
| FFD | Group × Initial MPP | 0.0464 | 2.0000 | 0.9770 |
| FFD | Frequency × Initial MPP | 0.0002 | 1.0000 | 0.9874 |
| FFD | Group × Final MPP | 1.0557 | 2.0000 | 0.5899 |
| FFD | Frequency × Final MPP | 0.0241 | 1.0000 | 0.8767 |
| FFD | Initial MPP × Final MPP | 0.0004 | 1.0000 | 0.9844 |
| FFD | Group × Frequency × Initial MPP | 2.0126 | 2.0000 | 0.3656 |
| FFD | Group × Frequency × Final MPP | 1.2335 | 2.0000 | 0.5397 |
| FFD | Group × Initial MPP × Final MPP | 0.7000 | 2.0000 | 0.7047 |
| FFD | Frequency × Initial MPP × Final MPP | 0.0914 | 1.0000 | 0.7625 |
| FFD | Group × Frequency × Initial MPP × Final MPP | 1.3342 | 2.0000 | 0.5132 |
| GD | Group | 108.8828 | 2.0000 | 0.0000 |
| GD | Frequency | 16.3287 | 1.0000 | 0.0001 |
| GD | Initial MPP | 0.3829 | 1.0000 | 0.5360 |
| GD | Final MPP | 0.0045 | 1.0000 | 0.9463 |
| GD | Group × Frequency | 9.2653 | 2.0000 | 0.0097 |
| GD | Group × Initial MPP | 6.0963 | 2.0000 | 0.0474 |
| GD | Frequency × Initial MPP | 3.9367 | 1.0000 | 0.0472 |
| GD | Group × Final MPP | 1.5279 | 2.0000 | 0.4658 |
| GD | Frequency × Final MPP | 1.7206 | 1.0000 | 0.1896 |
| GD | Initial MPP × Final MPP | 0.0011 | 1.0000 | 0.9736 |
| GD | Group × Frequency × Initial MPP | 0.8392 | 2.0000 | 0.6573 |
| GD | Group × Frequency × Final MPP | 1.2800 | 2.0000 | 0.5273 |
| GD | Group × Initial MPP × Final MPP | 7.9590 | 2.0000 | 0.0187 |
| GD | Frequency × Initial MPP × Final MPP | 0.7588 | 1.0000 | 0.3837 |
| GD | Group × Frequency × Initial MPP × Final MPP | 1.3459 | 2.0000 | 0.5102 |
| GPT | Group | 77.8346 | 2.0000 | 0.0000 |
| GPT | Frequency | 4.6180 | 1.0000 | 0.0316 |
| GPT | Initial MPP | 4.7141 | 1.0000 | 0.0299 |
| GPT | Final MPP | 1.3097 | 1.0000 | 0.2524 |
| GPT | Group × Frequency | 27.0553 | 2.0000 | 0.0000 |
| GPT | Group × Initial MPP | 4.0678 | 2.0000 | 0.1308 |
| GPT | Frequency × Initial MPP | 15.4903 | 1.0000 | 0.0001 |
| GPT | Group × Final MPP | 2.9481 | 2.0000 | 0.2290 |
| GPT | Frequency × Final MPP | 2.3924 | 1.0000 | 0.1219 |
| GPT | Initial MPP × Final MPP | 0.9669 | 1.0000 | 0.3255 |
| GPT | Group × Frequency × Initial MPP | 5.3202 | 2.0000 | 0.0699 |
| GPT | Group × Frequency × Final MPP | 1.8191 | 2.0000 | 0.4027 |
| GPT | Group × Initial MPP × Final MPP | 11.4698 | 2.0000 | 0.0032 |
| GPT | Frequency × Initial MPP × Final MPP | 4.6472 | 1.0000 | 0.0311 |
| GPT | Group × Frequency × Initial MPP × Final MPP | 4.7923 | 2.0000 | 0.0911 |
| TVT | Group | 80.8811 | 2.0000 | 0.0000 |
| TVT | Frequency | 6.0506 | 1.0000 | 0.0139 |
| TVT | Initial MPP | 0.5720 | 1.0000 | 0.4495 |
| TVT | Final MPP | 0.2122 | 1.0000 | 0.6450 |
| TVT | Group × Frequency | 22.1394 | 2.0000 | 0.0000 |
| TVT | Group × Initial MPP | 0.6581 | 2.0000 | 0.7196 |
| TVT | Frequency × Initial MPP | 5.8254 | 1.0000 | 0.0158 |
| TVT | Group × Final MPP | 2.7859 | 2.0000 | 0.2483 |
| TVT | Frequency × Final MPP | 1.8722 | 1.0000 | 0.1712 |
| TVT | Initial MPP × Final MPP | 0.0662 | 1.0000 | 0.7970 |
| TVT | Group × Frequency × Initial MPP | 0.4771 | 2.0000 | 0.7878 |
| TVT | Group × Frequency × Final MPP | 1.7504 | 2.0000 | 0.4168 |
| TVT | Group × Initial MPP × Final MPP | 5.9737 | 2.0000 | 0.0504 |
| TVT | Frequency × Initial MPP × Final MPP | 1.0553 | 1.0000 | 0.3043 |
| TVT | Group × Frequency × Initial MPP × Final MPP | 2.3299 | 2.0000 | 0.3119 |

# Supplementary Table S12. Cross-material model fit

| **Measure** | **N** | **Subjects** | **Items** | **Marginal R2** | **Conditional R2** | **Converged** | **AIC** |
| --- | --- | --- | --- | --- | --- | --- | --- |
| FFD | 4973.00 | 76.0000 | 69.0000 | 0.0383 | 0.1330 | 1.0000 | 3248.73 |
| GD | 4970.00 | 76.0000 | 69.0000 | 0.2556 | 0.3842 | 1.0000 | 7382.85 |
| GPT | 4981.00 | 76.0000 | 69.0000 | 0.2026 | 0.4799 | 1.0000 | 8363.05 |
| TVT | 4965.00 | 76.0000 | 69.0000 | 0.2519 | 0.3936 | 1.0000 | 7662.20 |

# Supplementary Table S13. Robustness analyses after excluding low-accuracy participants

| **Measure** | **Threshold** | **N** | **Subjects** | **Effect** | **Chi-square** | **df** | **p** |
| --- | --- | --- | --- | --- | --- | --- | --- |
| FFD | 0.7500 | 4603.00 | 70.0000 | Frequency × Initial MPP | 0.0002 | 1.0000 | 0.9876 |
| FFD | 0.7500 | 4603.00 | 70.0000 | Frequency × Initial MPP × Final MPP | 0.0897 | 1.0000 | 0.7645 |
| FFD | 0.7500 | 4603.00 | 70.0000 | Group × Frequency × Initial MPP × Final MPP | 0.6470 | 2.0000 | 0.7236 |
| FFD | 0.8000 | 4337.00 | 66.0000 | Frequency × Initial MPP | 0.0003 | 1.0000 | 0.9871 |
| FFD | 0.8000 | 4337.00 | 66.0000 | Frequency × Initial MPP × Final MPP | 0.0897 | 1.0000 | 0.7646 |
| FFD | 0.8000 | 4337.00 | 66.0000 | Group × Frequency × Initial MPP × Final MPP | 1.7390 | 2.0000 | 0.4192 |
| GD | 0.7500 | 4598.00 | 70.0000 | Frequency × Initial MPP | 4.0249 | 1.0000 | 0.0448 |
| GD | 0.7500 | 4598.00 | 70.0000 | Frequency × Initial MPP × Final MPP | 0.7714 | 1.0000 | 0.3798 |
| GD | 0.7500 | 4598.00 | 70.0000 | Group × Frequency × Initial MPP × Final MPP | 2.2936 | 2.0000 | 0.3176 |
| GD | 0.8000 | 4331.00 | 66.0000 | Frequency × Initial MPP | 4.0938 | 1.0000 | 0.0430 |
| GD | 0.8000 | 4331.00 | 66.0000 | Frequency × Initial MPP × Final MPP | 0.7920 | 1.0000 | 0.3735 |
| GD | 0.8000 | 4331.00 | 66.0000 | Group × Frequency × Initial MPP × Final MPP | 1.3993 | 2.0000 | 0.4968 |
| GPT | 0.7500 | 4608.00 | 70.0000 | Frequency × Initial MPP | 15.8902 | 1.0000 | 0.0001 |
| GPT | 0.7500 | 4608.00 | 70.0000 | Frequency × Initial MPP × Final MPP | 4.7824 | 1.0000 | 0.0288 |
| GPT | 0.7500 | 4608.00 | 70.0000 | Group × Frequency × Initial MPP × Final MPP | 9.0960 | 2.0000 | 0.0106 |
| GPT | 0.8000 | 4344.00 | 66.0000 | Frequency × Initial MPP | 15.8178 | 1.0000 | 0.0001 |
| GPT | 0.8000 | 4344.00 | 66.0000 | Frequency × Initial MPP × Final MPP | 4.7980 | 1.0000 | 0.0285 |
| GPT | 0.8000 | 4344.00 | 66.0000 | Group × Frequency × Initial MPP × Final MPP | 8.5392 | 2.0000 | 0.0140 |
| TVT | 0.7500 | 4593.00 | 70.0000 | Frequency × Initial MPP | 5.9179 | 1.0000 | 0.0150 |
| TVT | 0.7500 | 4593.00 | 70.0000 | Frequency × Initial MPP × Final MPP | 1.0725 | 1.0000 | 0.3004 |
| TVT | 0.7500 | 4593.00 | 70.0000 | Group × Frequency × Initial MPP × Final MPP | 5.4899 | 2.0000 | 0.0643 |
| TVT | 0.8000 | 4325.00 | 66.0000 | Frequency × Initial MPP | 5.8828 | 1.0000 | 0.0153 |
| TVT | 0.8000 | 4325.00 | 66.0000 | Frequency × Initial MPP × Final MPP | 1.0660 | 1.0000 | 0.3018 |
| TVT | 0.8000 | 4325.00 | 66.0000 | Group × Frequency × Initial MPP × Final MPP | 5.2499 | 2.0000 | 0.0724 |

# Supplementary Table S14. Full fixed-effect estimates for FFD

| **Measure** | **Fixed effect** | **b** | **SE** | **z** | **p** | **CI_low** | **CI_high** |
| --- | --- | --- | --- | --- | --- | --- | --- |
| FFD | Intercept | 5.4365 | 0.0285 | 190.7658 | 0.0000 | 5.3806 | 5.4923 |
| FFD | Advanced vs. Native | 0.0863 | 0.0432 | 1.9993 | 0.0456 | 0.0017 | 0.1709 |
| FFD | Intermediate vs. Native | 0.1342 | 0.0441 | 3.0426 | 0.0023 | 0.0477 | 0.2206 |
| FFD | Low vs. High frequency | 0.0333 | 0.0302 | 1.1013 | 0.2708 | -0.0260 | 0.0925 |
| FFD | Low vs. High initial MPP | -0.0052 | 0.0289 | -0.1787 | 0.8582 | -0.0617 | 0.0514 |
| FFD | Low vs. High final MPP | 0.0020 | 0.0288 | 0.0678 | 0.9460 | -0.0545 | 0.0584 |
| FFD | Advanced vs. Native x Low vs. High frequency | -0.0019 | 0.0455 | -0.0420 | 0.9665 | -0.0911 | 0.0873 |
| FFD | Intermediate vs. Native x Low vs. High frequency | 0.0719 | 0.0467 | 1.5380 | 0.1241 | -0.0197 | 0.1635 |
| FFD | Advanced vs. Native x Low vs. High initial MPP | 0.0054 | 0.0431 | 0.1244 | 0.9010 | -0.0790 | 0.0898 |
| FFD | Intermediate vs. Native x Low vs. High initial MPP | 0.0094 | 0.0443 | 0.2127 | 0.8316 | -0.0773 | 0.0962 |
| FFD | Low vs. High frequency x Low vs. High initial MPP | 0.0007 | 0.0418 | 0.0158 | 0.9874 | -0.0812 | 0.0825 |
| FFD | Advanced vs. Native x Low vs. High final MPP | -0.0209 | 0.0431 | -0.4836 | 0.6287 | -0.1054 | 0.0637 |
| FFD | Intermediate vs. Native x Low vs. High final MPP | 0.0268 | 0.0442 | 0.6064 | 0.5443 | -0.0599 | 0.1135 |
| FFD | Low vs. High frequency x Low vs. High final MPP | -0.0066 | 0.0424 | -0.1552 | 0.8767 | -0.0896 | 0.0764 |
| FFD | Low vs. High initial MPP x Low vs. High final MPP | -0.0008 | 0.0402 | -0.0195 | 0.9844 | -0.0796 | 0.0780 |
| FFD | Advanced vs. Native x Low vs. High frequency x Low vs. High initial MPP | -0.0107 | 0.0626 | -0.1701 | 0.8649 | -0.1334 | 0.1121 |
| FFD | Intermediate vs. Native x Low vs. High frequency x Low vs. High initial MPP | -0.0867 | 0.0645 | -1.3437 | 0.1790 | -0.2130 | 0.0397 |
| FFD | Advanced vs. Native x Low vs. High frequency x Low vs. High final MPP | -0.0041 | 0.0635 | -0.0645 | 0.9486 | -0.1285 | 0.1203 |
| FFD | Intermediate vs. Native x Low vs. High frequency x Low vs. High final MPP | -0.0670 | 0.0652 | -1.0276 | 0.3042 | -0.1949 | 0.0608 |
| FFD | Advanced vs. Native x Low vs. High initial MPP x Low vs. High final MPP | 0.0253 | 0.0602 | 0.4202 | 0.6743 | -0.0927 | 0.1432 |
| FFD | Intermediate vs. Native x Low vs. High initial MPP x Low vs. High final MPP | -0.0290 | 0.0618 | -0.4691 | 0.6390 | -0.1501 | 0.0921 |
| FFD | Low vs. High frequency x Low vs. High initial MPP x Low vs. High final MPP | 0.0180 | 0.0595 | 0.3023 | 0.7625 | -0.0987 | 0.1347 |
| FFD | Advanced vs. Native x Low vs. High frequency x Low vs. High initial MPP x Low vs. High final MPP | 0.0475 | 0.0895 | 0.5315 | 0.5951 | -0.1278 | 0.2229 |
| FFD | Intermediate vs. Native x Low vs. High frequency x Low vs. High initial MPP x Low vs. High final MPP | 0.1061 | 0.0919 | 1.1545 | 0.2483 | -0.0741 | 0.2864 |

# Supplementary Table S14. Full fixed-effect estimates for GD

| **Measure** | **Fixed effect** | **b** | **SE** | **z** | **p** | **CI_low** | **CI_high** |
| --- | --- | --- | --- | --- | --- | --- | --- |
| GD | Intercept | 5.5637 | 0.0467 | 119.1913 | 0.0000 | 5.4723 | 5.6552 |
| GD | Advanced vs. Native | 0.4277 | 0.0707 | 6.0526 | 0.0000 | 0.2892 | 0.5662 |
| GD | Intermediate vs. Native | 0.7403 | 0.0721 | 10.2724 | 0.0000 | 0.5990 | 0.8815 |
| GD | Low vs. High frequency | 0.1852 | 0.0458 | 4.0409 | 0.0001 | 0.0954 | 0.2750 |
| GD | Low vs. High initial MPP | 0.0272 | 0.0440 | 0.6188 | 0.5360 | -0.0590 | 0.1135 |
| GD | Low vs. High final MPP | 0.0029 | 0.0438 | 0.0674 | 0.9463 | -0.0828 | 0.0887 |
| GD | Advanced vs. Native x Low vs. High frequency | 0.1679 | 0.0688 | 2.4408 | 0.0147 | 0.0331 | 0.3028 |
| GD | Intermediate vs. Native x Low vs. High frequency | -0.0406 | 0.0703 | -0.5780 | 0.5632 | -0.1784 | 0.0971 |
| GD | Advanced vs. Native x Low vs. High initial MPP | -0.0086 | 0.0653 | -0.1320 | 0.8950 | -0.1366 | 0.1194 |
| GD | Intermediate vs. Native x Low vs. High initial MPP | -0.1526 | 0.0672 | -2.2706 | 0.0232 | -0.2842 | -0.0209 |
| GD | Low vs. High frequency x Low vs. High initial MPP | -0.1256 | 0.0633 | -1.9841 | 0.0472 | -0.2496 | -0.0015 |
| GD | Advanced vs. Native x Low vs. High final MPP | -0.0409 | 0.0653 | -0.6272 | 0.5306 | -0.1689 | 0.0870 |
| GD | Intermediate vs. Native x Low vs. High final MPP | -0.0826 | 0.0671 | -1.2321 | 0.2179 | -0.2140 | 0.0488 |
| GD | Low vs. High frequency x Low vs. High final MPP | -0.0846 | 0.0645 | -1.3117 | 0.1896 | -0.2111 | 0.0418 |
| GD | Low vs. High initial MPP x Low vs. High final MPP | 0.0020 | 0.0612 | 0.0331 | 0.9736 | -0.1180 | 0.1220 |
| GD | Advanced vs. Native x Low vs. High frequency x Low vs. High initial MPP | -0.0566 | 0.0948 | -0.5976 | 0.5501 | -0.2423 | 0.1291 |
| GD | Intermediate vs. Native x Low vs. High frequency x Low vs. High initial MPP | 0.0356 | 0.0973 | 0.3658 | 0.7145 | -0.1551 | 0.2262 |
| GD | Advanced vs. Native x Low vs. High frequency x Low vs. High final MPP | 0.1092 | 0.0966 | 1.1306 | 0.2582 | -0.0801 | 0.2984 |
| GD | Intermediate vs. Native x Low vs. High frequency x Low vs. High final MPP | 0.0449 | 0.0986 | 0.4560 | 0.6484 | -0.1482 | 0.2381 |
| GD | Advanced vs. Native x Low vs. High initial MPP x Low vs. High final MPP | 0.1905 | 0.0913 | 2.0875 | 0.0368 | 0.0116 | 0.3694 |
| GD | Intermediate vs. Native x Low vs. High initial MPP x Low vs. High final MPP | 0.2458 | 0.0938 | 2.6190 | 0.0088 | 0.0618 | 0.4297 |
| GD | Low vs. High frequency x Low vs. High initial MPP x Low vs. High final MPP | 0.0788 | 0.0905 | 0.8711 | 0.3837 | -0.0985 | 0.2561 |
| GD | Advanced vs. Native x Low vs. High frequency x Low vs. High initial MPP x Low vs. High final MPP | -0.1575 | 0.1358 | -1.1598 | 0.2461 | -0.4237 | 0.1087 |
| GD | Intermediate vs. Native x Low vs. High frequency x Low vs. High initial MPP x Low vs. High final MPP | -0.0733 | 0.1390 | -0.5274 | 0.5979 | -0.3458 | 0.1991 |

# Supplementary Table S14. Full fixed-effect estimates for GPT

| **Measure** | **Fixed effect** | **b** | **SE** | **z** | **p** | **CI_low** | **CI_high** |
| --- | --- | --- | --- | --- | --- | --- | --- |
| GPT | Intercept | 5.8476 | 0.0512 | 114.2645 | 0.0000 | 5.7473 | 5.9479 |
| GPT | Advanced vs. Native | 0.2594 | 0.0777 | 3.3398 | 0.0008 | 0.1072 | 0.4116 |
| GPT | Intermediate vs. Native | 0.6985 | 0.0793 | 8.8109 | 0.0000 | 0.5431 | 0.8539 |
| GPT | Low vs. High frequency | 0.1078 | 0.0502 | 2.1490 | 0.0316 | 0.0095 | 0.2061 |
| GPT | Low vs. High initial MPP | 0.1056 | 0.0486 | 2.1712 | 0.0299 | 0.0103 | 0.2010 |
| GPT | Low vs. High final MPP | 0.0557 | 0.0487 | 1.1444 | 0.2524 | -0.0397 | 0.1512 |
| GPT | Advanced vs. Native x Low vs. High frequency | 0.3348 | 0.0752 | 4.4504 | 0.0000 | 0.1874 | 0.4823 |
| GPT | Intermediate vs. Native x Low vs. High frequency | -0.0386 | 0.0772 | -0.5000 | 0.6171 | -0.1900 | 0.1128 |
| GPT | Advanced vs. Native x Low vs. High initial MPP | -0.0686 | 0.0725 | -0.9464 | 0.3440 | -0.2107 | 0.0735 |
| GPT | Intermediate vs. Native x Low vs. High initial MPP | -0.1506 | 0.0747 | -2.0156 | 0.0438 | -0.2971 | -0.0042 |
| GPT | Low vs. High frequency x Low vs. High initial MPP | -0.2745 | 0.0697 | -3.9358 | 0.0001 | -0.4112 | -0.1378 |
| GPT | Advanced vs. Native x Low vs. High final MPP | -0.0588 | 0.0726 | -0.8094 | 0.4183 | -0.2010 | 0.0835 |
| GPT | Intermediate vs. Native x Low vs. High final MPP | -0.1276 | 0.0744 | -1.7159 | 0.0862 | -0.2734 | 0.0182 |
| GPT | Low vs. High frequency x Low vs. High final MPP | -0.1097 | 0.0709 | -1.5468 | 0.1219 | -0.2487 | 0.0293 |
| GPT | Low vs. High initial MPP x Low vs. High final MPP | -0.0670 | 0.0682 | -0.9833 | 0.3255 | -0.2006 | 0.0666 |
| GPT | Advanced vs. Native x Low vs. High frequency x Low vs. High initial MPP | 0.0299 | 0.1045 | 0.2861 | 0.7748 | -0.1750 | 0.2348 |
| GPT | Intermediate vs. Native x Low vs. High frequency x Low vs. High initial MPP | 0.2351 | 0.1075 | 2.1871 | 0.0287 | 0.0244 | 0.4459 |
| GPT | Advanced vs. Native x Low vs. High frequency x Low vs. High final MPP | 0.0208 | 0.1061 | 0.1964 | 0.8443 | -0.1872 | 0.2288 |
| GPT | Intermediate vs. Native x Low vs. High frequency x Low vs. High final MPP | 0.1399 | 0.1087 | 1.2866 | 0.1982 | -0.0732 | 0.3530 |
| GPT | Advanced vs. Native x Low vs. High initial MPP x Low vs. High final MPP | 0.3080 | 0.1016 | 3.0311 | 0.0024 | 0.1089 | 0.5072 |
| GPT | Intermediate vs. Native x Low vs. High initial MPP x Low vs. High final MPP | 0.2801 | 0.1042 | 2.6871 | 0.0072 | 0.0758 | 0.4844 |
| GPT | Low vs. High frequency x Low vs. High initial MPP x Low vs. High final MPP | 0.2150 | 0.0997 | 2.1557 | 0.0311 | 0.0195 | 0.4104 |
| GPT | Advanced vs. Native x Low vs. High frequency x Low vs. High initial MPP x Low vs. High final MPP | -0.2371 | 0.1498 | -1.5827 | 0.1135 | -0.5306 | 0.0565 |
| GPT | Intermediate vs. Native x Low vs. High frequency x Low vs. High initial MPP x Low vs. High final MPP | -0.3146 | 0.1536 | -2.0478 | 0.0406 | -0.6157 | -0.0135 |

# Supplementary Table S14. Full fixed-effect estimates for TVT

| **Measure** | **Fixed effect** | **b** | **SE** | **z** | **p** | **CI_low** | **CI_high** |
| --- | --- | --- | --- | --- | --- | --- | --- |
| TVT | Intercept | 5.8849 | 0.0548 | 107.3825 | 0.0000 | 5.7775 | 5.9924 |
| TVT | Advanced vs. Native | 0.3826 | 0.0834 | 4.5881 | 0.0000 | 0.2192 | 0.5461 |
| TVT | Intermediate vs. Native | 0.7599 | 0.0849 | 8.9509 | 0.0000 | 0.5935 | 0.9263 |
| TVT | Low vs. High frequency | 0.1152 | 0.0468 | 2.4598 | 0.0139 | 0.0234 | 0.2070 |
| TVT | Low vs. High initial MPP | 0.0340 | 0.0450 | 0.7563 | 0.4495 | -0.0541 | 0.1221 |
| TVT | Low vs. High final MPP | 0.0206 | 0.0448 | 0.4607 | 0.6450 | -0.0672 | 0.1085 |
| TVT | Advanced vs. Native x Low vs. High frequency | 0.2516 | 0.0705 | 3.5708 | 0.0004 | 0.1135 | 0.3898 |
| TVT | Intermediate vs. Native x Low vs. High frequency | -0.0878 | 0.0720 | -1.2194 | 0.2227 | -0.2290 | 0.0533 |
| TVT | Advanced vs. Native x Low vs. High initial MPP | 0.0287 | 0.0670 | 0.4289 | 0.6680 | -0.1026 | 0.1601 |
| TVT | Intermediate vs. Native x Low vs. High initial MPP | -0.0298 | 0.0690 | -0.4323 | 0.6656 | -0.1650 | 0.1054 |
| TVT | Low vs. High frequency x Low vs. High initial MPP | -0.1562 | 0.0647 | -2.4136 | 0.0158 | -0.2830 | -0.0293 |
| TVT | Advanced vs. Native x Low vs. High final MPP | 0.0163 | 0.0670 | 0.2428 | 0.8081 | -0.1151 | 0.1476 |
| TVT | Intermediate vs. Native x Low vs. High final MPP | -0.0947 | 0.0686 | -1.3805 | 0.1674 | -0.2293 | 0.0398 |
| TVT | Low vs. High frequency x Low vs. High final MPP | -0.0902 | 0.0659 | -1.3683 | 0.1712 | -0.2194 | 0.0390 |
| TVT | Low vs. High initial MPP x Low vs. High final MPP | 0.0161 | 0.0628 | 0.2573 | 0.7970 | -0.1069 | 0.1392 |
| TVT | Advanced vs. Native x Low vs. High frequency x Low vs. High initial MPP | -0.0508 | 0.0971 | -0.5235 | 0.6006 | -0.2412 | 0.1395 |
| TVT | Intermediate vs. Native x Low vs. High frequency x Low vs. High initial MPP | 0.0180 | 0.0997 | 0.1801 | 0.8571 | -0.1774 | 0.2133 |
| TVT | Advanced vs. Native x Low vs. High frequency x Low vs. High final MPP | 0.0609 | 0.0987 | 0.6172 | 0.5371 | -0.1325 | 0.2544 |
| TVT | Intermediate vs. Native x Low vs. High frequency x Low vs. High final MPP | 0.1334 | 0.1009 | 1.3223 | 0.1861 | -0.0644 | 0.3312 |
| TVT | Advanced vs. Native x Low vs. High initial MPP x Low vs. High final MPP | 0.2178 | 0.0938 | 2.3227 | 0.0202 | 0.0340 | 0.4017 |
| TVT | Intermediate vs. Native x Low vs. High initial MPP x Low vs. High final MPP | 0.1631 | 0.0961 | 1.6985 | 0.0894 | -0.0251 | 0.3514 |
| TVT | Low vs. High frequency x Low vs. High initial MPP x Low vs. High final MPP | 0.0952 | 0.0927 | 1.0273 | 0.3043 | -0.0865 | 0.2769 |
| TVT | Advanced vs. Native x Low vs. High frequency x Low vs. High initial MPP x Low vs. High final MPP | -0.2062 | 0.1391 | -1.4820 | 0.1384 | -0.4788 | 0.0665 |
| TVT | Intermediate vs. Native x Low vs. High frequency x Low vs. High initial MPP x Low vs. High final MPP | -0.1385 | 0.1426 | -0.9709 | 0.3316 | -0.4180 | 0.1411 |
